# Pluronics-Based Drug Delivery Systems for Flavonoids Anticancer Treatment

**DOI:** 10.3390/gels9020143

**Published:** 2023-02-08

**Authors:** Sylwia Ronka, Aleksandra Kowalczyk, Dagmara Baczyńska, Anna K. Żołnierczyk

**Affiliations:** 1 Department of Polymer Engineering and Technology, Faculty of Chemistry, Wrocław University of Science and Technology, Wybrzeże Wyspiańskiego 27, 50-370 Wrocław, Poland; 2 Department of Molecular and Cellular Biology, Faculty of Pharmacy with Division of Laboratory Diagnostics, Wrocław Medical University, Borowska 211A, 50-556 Wrocław, Poland; 3 Department of Food Chemistry and Biocatalysis, Faculty of Biotechnology and Food Science, Wrocław University of Environmental and Life Sciences, Norwida 25, 50-375 Wrocław, Poland

**Keywords:** amphiphilic copolymers, flavonoids, cancer therapy, drug delivery systems, nanostructures

## Abstract

This research concerns the investigation of the preparation of polymeric nanocarriers containing a flavonoid—naringenin, xanthohumol or isoxanthohumol—based on Pluronics by the thin-film formation method. The size of the formed micelles and their stability upon dilution were evaluated using Dynamic light scattering (DLS) analysis; the high values of the drug loading and the encapsulation efficiency confirmed that the proposed systems of flavonoids delivery consisting of Pluronic P123 and F127 nanomicelles could effectively distribute the drug into tumour tissues, which makes these nanocarriers ideal candidates for passive targeting of cancer cells by the enhanced permeation and retention (EPR) effect. The in vitro cytotoxicity of proposed flavonoids in the Pluronic formulations was investigated by the SRB assay with human colon cancer cells. We designed mixed polymeric micelles, which was a successful drug delivery system for the case of naringenin not being able to enhance the bioavailability and cytotoxic activity of xanthohumol and isoxanthohumol. Furthermore, it was observed that the higher amount of polymer in the formulation achieved better cytotoxic activity.

## 1. Introduction

Many clinical studies revealed that conventional anticancer agents’ therapeutic efficacy is insufficient, despite promising in vitro experiments. The reduced efficacy of the chemotherapy arises from poor solubility of compounds in an aqueous environment, inappropriate pharmacokinetic characteristics, insufficient penetration of tumour vessels, activation of multidrug resistance (MDR) in cancer cells, and toxic effects towards normal cells. Therefore, several approaches have been applied to overcome these problems, e.g., investigating new alternative anticancer agents, and developing targeted nanoscale drug delivery systems [1].

Compounds of natural origin are of great interest to researchers, due to their diverse biological activities. Among them, natural flavonoids and their synthetic derivatives possess high anticancer abilities. Naringenin (NG) and prenylated flavonoids such as xanthoumol (XH) and isoxanthohumol (IXH) are very attractive compounds because of their high bioactivity, accompanied by easy and inexpensive access [2,3,4]. Naringenin exerts an anti-inflammatory effect by inhibiting the production of nitric oxide and prostaglandin E2 [5]. In addition, antiproliferative and proapoptotic effects of naringenin have been confirmed in many human cancer cell lines, i.e., breast, colon, uterus, melanoma, and prostate. Furthermore, orally administered naringenin inhibits the metastasis process after breast cancer surgery by regulating host immunity. It is noteworthy that naringenin has low toxicity to normal cells [6]. Similarly, xanthohumol has a wide spectrum of chemoprotective and anticancer properties, including induction of carcinogen-detoxifying enzymes, metabolic inactivation of procarcinogens, and inhibition of tumour growth. The proapoptotic and antiproliferative activities of xanthohumol and isoxanthohumol have been reported against different cancer cells, such as chronic lymphocytic leukemia, prostate, colon, breast, and laryngeal [7,8,9]. However, the use of these compounds in clinical applications is still limited due to their poor water solubility, sensitivity to high temperatures and acidity, and also their low bioavailability (gastrointestinal absorption usually does not exceed 10%) [5].

There are many investigations to develop the best drug nanocarriers to improve drug pharmacokinetic features, increase delivery efficiency into cells, and decrease drug-related side effects [10,11,12,13]. Polymeric micelles especially seem attractive for such applications; some have been tested in clinical trials. Polymeric micelles have low critical micellisation concentration values (CMC), so they can retain drug molecules for longer, even after dilution in systemic fluids. Furthermore, they exhibit suitable micelle sizes (usually 20–200 nm), high encapsulation capacities, ease of preparation, and a large possibility of system modifications [14,15,16,17,18]. To better review the properties of polymeric micelles and their applications, we recommend [19,20,21,22].

Among various polymers approved for medical applications, Pluronics have a growing interest from researchers. Pluronics are amphiphilic triblock copolymers of polyethylene oxide (PEO) and polypropylene oxide (PPO)—(PEO-PPO-PEO copolymer). They have been widely investigated as anticancer drug carriers, and have yielded promising results [23,24,25,26,27]. The efficacy of Pluronic copolymers as components of drug delivery systems arises from their ability to modify drug pharmacokinetic parameters, such as water solubility and prevention of adverse pH, and their potential role as modifiers of the biological response. Numerous studies have shown that Pluronics can decrease the MDR effects of drugs by inhibiting P-glycoproteins and other drug efflux transporters. Other possible mechanisms of anticancer action assume that Pluronics can modify the microviscosity of the cellular membrane and inhibit respiration in mitochondria, resulting in the depletion of ATP [25]. The chemical and physical properties of Pluronic copolymers, such as CMC and micelle shape, depend mainly on the ratio between hydrophobic polypropylene oxide and hydrophilic polyethylene oxide blocks content, which can vary from 1:9 to 8:2.

Previously, the effects of encapsulations of only some flavonoids (myricentin, apigenin, genistein) with Pluronics were investigated in different cancer cell lines, which confirmed better cellular uptake and cytotoxic effect of drugs on cells, and indicated increased solubility of complexes in water solutions [28,29,30]. Here, we optimised the composition and concentration of Pluronics with other highly hydrophobic flavonoids: NG, XH, and IXH, and tested the utility of such a delivery system in a model of anticancer therapy with human colon adenocarcinoma cells HT29. The size of the formed micelles and their stability upon dilution were evaluated to assess their potential for targeting tumour cells. Furthermore, the cytotoxic effects between encapsulated flavonoids and free forms were compared.

## 2. Results and Discussion

Two types of Pluronics, P123 and F127, were used in flavonoid encapsulation studies. Pluronic P123 is an amphiphilic block copolymer with a structure PEO–PPO–PEO, and is commonly used in pharmaceutical applications. As the hydrophobic PPO part constitutes as much as 70% of the molecule, this enables it to solubilise sufficient amounts of poorly water-soluble drugs; the hydrophilic PEO part is responsible for maintaining the micelles’ stability. However, the fact that there is only 30% of the PEO blocks in the molecule leads to thin micelle shell formation, and as a result, poor dispersibility. Once the ratio of the hydrophilic and hydrophobic part in Pluronic P123 chain is unbalanced, instability of the micelles may occur. In order to avoid destabilisation, it is recommended to make some modifications to single-type P123 micelles, such as the introduction of other amphiphilic polymers with larger hydrophilic parts. This results in mixed micelles formations, which are known for their numerous advantages [29]. Pluronic F127 has gained significant attention, thanks to its thermosensitivity and wide range of possible biomedical applications. The amount of PPE and PEO blocks in the molecule of Pluronic F127 is opposite to Pluronic P123—30% of PPO and 70% of PEO—which leads to different properties of Pluronic F127, and makes it possible to take advantage of the properties of mixed micellar systems comprising these two Pluronics.

### 2.1. Visual Assessment

**Figure 1:**
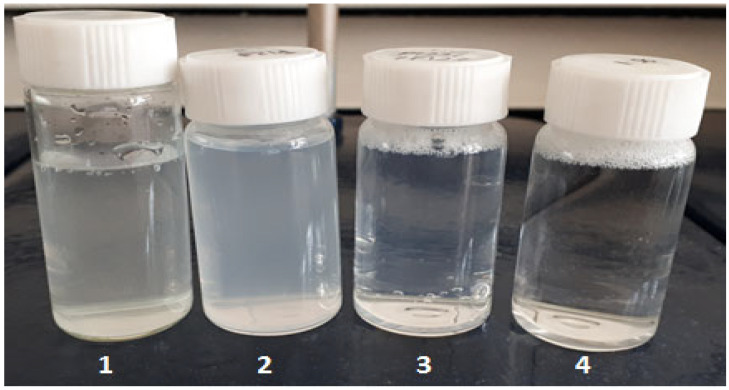
Comparison of the naringenin suspension (0.125 mg/mL) (**1**), and naringenin-loaded micelles solutions in water with various drug to polymer ratios: Pluronic P123 micelles 1:5 C = 0.750 mg/mL (**2**), Pluronic P123 and F127 mixed micelles 1:5 C = 0.750 mg/mL (**3**), Pluronic P123 and F127 mixed micelles 1:50 C = 6.375 mg/mL (**4**).

As shown in Figure 1, after the dissolution of crude naringenin in distilled water, a suspension of insoluble particles (1) was obtained, while naringenin-loaded micelles (2, 3, 4) formed homogeneous solutions. The appearance of micelles ranged from clear, when the drug to polymer ratios were high (4), to opaque in the case of Pluronic P123 1:5 (2). The concentration of naringenin in all samples was uniform. 

**Figure 2:**
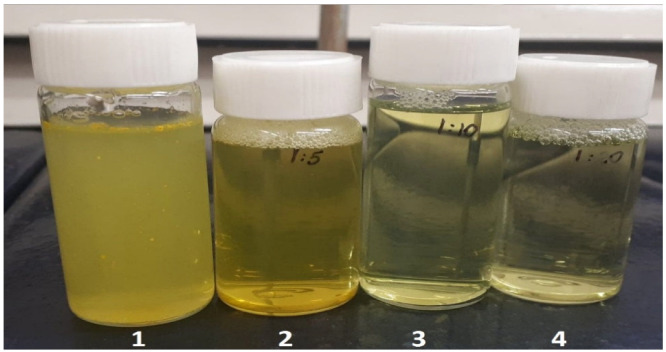
Comparison of the xanthohumol suspension (0.125 mg/mL) (**1**), and xanthohumol-loaded micelles solutions in water with various drug to polymer ratios: Pluronic P123 and F127 mixed micelles 1:5 C = 0.75 mg/mL (**2**), 1:10 C = 1.375 mg/mL (**3**) and 1:20 C = 2.625 mg/mL (**4**).

The dissolution of xanthohumol in water was even harder than in the case of naringenin, which corresponded to the values of their water solubilities of 0.30 mg/L and 0.0013 mg/mL, respectively. Finally, a suspension of the undissolved particles (Figure 2—(1)) was obtained. As shown in Figure 2, xanthohumol-loaded micelles (2, 3, 4) formed homogeneous solutions. The appearance of xanthohumol-loaded micelles ranged from clear, when drug-polymer ratios were high (4), to opaque in the case of 1:5 (2). The concentration of xanthohumol in all samples was uniform.

The opaque appearance of samples with a drug to polymer ratio of 1:5 may be a result of the incomplete coverage of the drug by a polymer. For the higher polymer amount in the samples, the solutions were observed to be clearer, and dissolved easily. This may have occurred because the higher amount of polymer was sufficient to solubilise the total amount of drug encapsulated in the sample.

### 2.2. Encapsulation Efficiency and Drug Loading

The main factors that influence drug loading and encapsulation efficiency are the nature and length of the core-forming block, as well as the total molecular weight of the copolymer. Moreover, in the case of the preparation of mixed micelles, a similar length of hydrophobic blocks is required to provide copolymers compatibility, and hence mixed micelle stability. The values of drug loading (DL) and encapsulation efficiency (EE) of flavonoids into Pluronic micelles are summarised in Table 1 and Table 2. 

All formulations resulted in very high drug loadings, with encapsulation efficiencies ranging from 93.5 to 100%. The ratio of the flavonoid to the polymer had no impact on either drug loading or encapsulation efficiency. The thin-film formation method usually provides high encapsulation efficiencies, and that was also achieved in this case.

### 2.3. Particle Size Analysis

**Figure 3:**
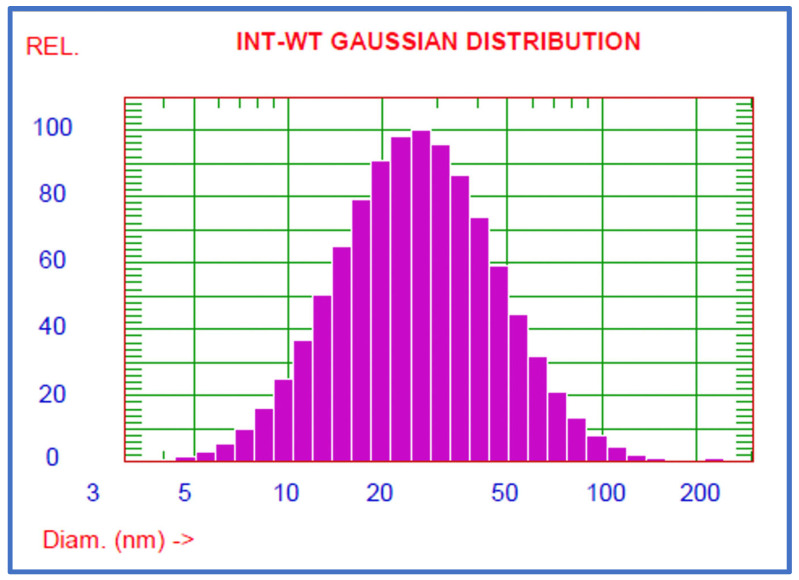
Size distribution of xanthohumol-loaded polymeric micelles based on Pluronics P123 and F127 (ratio 1:10). Mean diameter 30.5 ± 18.0 nm.

The size of the nanocarrier is an important parameter for its in vivo performance and its ability to accumulate in the target areas, such as tumour tissues. Taking into consideration both pharmacodynamic and pharmacokinetic aspects, it was estimated that the advantageous size of an anticancer drug carrier should be in the range between 20 and 200 nm. The results of particle size and distribution measurement using the DLS technique are shown in Table 3 and Table 4. An example of the plot obtained from the DLS size measuring equipment, which presents the Gaussian distribution of the micelles size, is shown in Figure 3.

In regards to naringenin, the mean diameter of obtained nanomicelles ranged from 27.5 to 229.4 nm, depending on the type of Pluronic used and its content in the formulation. Smaller particles (27.5–38.2 nm) were obtained when Pluronic F127 was used as an encapsulation agent. The samples that showed the biggest dimensions were those with the lowest (1:5) ratio of naringenin to polymer.

The relative stability of particle size was observed in the case of mixed micelles of Pluronics P123 and F127. This trend was even more noticeable in xanthohumol-loaded nanomicelles, which were characterised by a uniform size of the particles—30.5 nm—regardless of the flavonoid to Pluronic ratio.

An investigation of micelle stability on dilution was conducted for naringenin-loaded nanomicelles (Table 5). It was observed that mixed micelles were less susceptible to destabilisation upon dilution than single-type micelles. This observation has confirmed the theory found in the literature [30]. At the most critical point, their size has changed by 17.0 nm from 138.1 nm to 155.1 nm, whereas in the case of Pluronic P123 single-type micelles, the difference was 193.1 nm (from 229.4 to 36.3 nm), which indicated destabilisation of the micelles.

The calculated values of the polydispersity index (see Table 3 and Table 4) showed that the size distribution of the obtained nanoparticles was quite diverse, which is a disadvantageous factor, and may require further optimisation at a later stage of research. Zeta potential measurement, which was used to investigate the potential between droplet surface and dispersing liquid medium, showed relatively neutral values ranging from −8.88 to 8.13 mV. This analysis was used to estimate the surface charge of the micelles in the dispersion medium, and thus its stability. Overall, however, the sizes of obtained nanomicelles were in the range between 10 and 200 nm, which allow the particles to overcome renal clearance and capturing by RES, as well as lead to their passive accumulation in tumours through the EPR effect. The efficient drug delivery of such small particles is possible by oral administration and intravenous injection [31].

### 2.4. Differential Scanning Calorimetry

Thermograms of free naringenin, Pluronic F127, and nanomicellar formulation of naringenin encapsulated in Pluronic F127 1:10 were detected via DSC analysis. 

**Figure 4:**
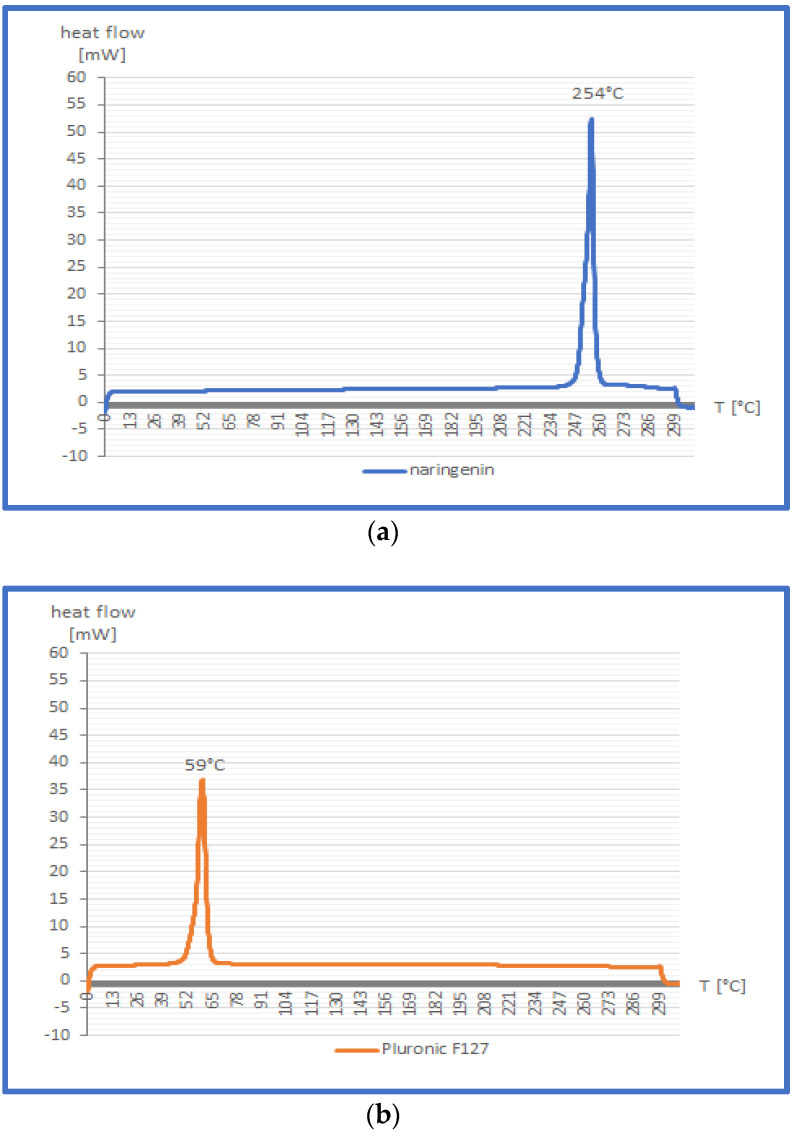

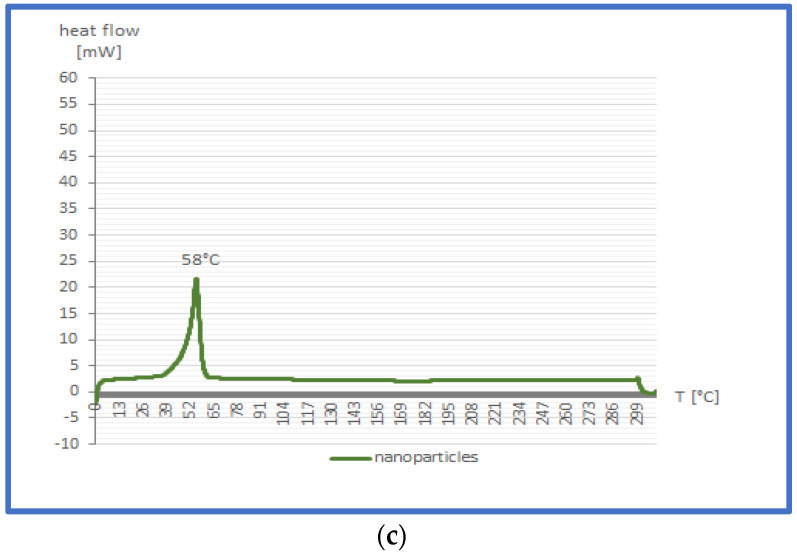
Results of DSC analysis of crude naringenin (**a**), Pluronic F127 (**b**) and naringenin–Pluronic F127 1:10 nanoparticles (**c**).

As recorded in the DCS curves (Figure 4a–c), the melting point of free naringenin was approximately 254 °C, whereas the narrow and sharp Pluronic F127 melting peak was found at about 59 °C. Naringenin-loaded nanomicelles exhibited an endothermic peak at nearly the same temperature as Pluronic—namely 58 °C; however, the peak’s shape was broader. The melting transition peak of naringenin disappeared in the case of the nanomicellar formulation. In the literature [32], the disappearance of the endothermic peak of the drug in a nanocarrier indicates its amorphous state, and consequently means that the flavonoid has been fully encapsulated into polymer [31].

### 2.5. In Vitro Release of Flavonoids from Pluronic Micelles

**Figure 5:**
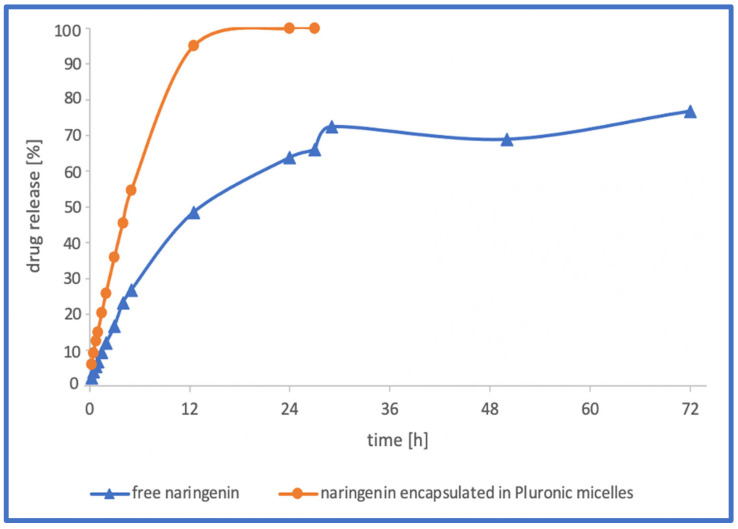
In vitro release profile of naringenin-loaded P123/F127 Pluronic micelles (ratio 1:5) and free naringenin.

The drug release profiles of naringenin were determined via the dialysis method under physiological conditions (37 °C, pH = 7.4). The experiment was carried out in a release medium containing PBS (phosphate buffer saline) and 20% (*v*/*v*) ethanol for 72 h. The resulting release profiles—percent of drug release versus time—of free naringenin and naringenin encapsulated in Pluronic-based polymeric micelles (ratio 1:5) are shown in Figure 5.

Encapsulation in polymeric micelles enabled a complete (100%) release of naringenin after 16 h, while only 55% of non-encapsulated naringenin was dissolved after the same time. The maximum possible concentration of non-encapsulated naringenin, 77%, was achieved after 72 h. Moreover, two phases could be distinguished in the drug release profile of naringenin loaded in polymeric micelles. The initial rapid release observed during the first 4.5 h was 50% of the encapsulated naringenin. That phase was followed by more sustained release behaviour maintained for the next 11 h of the experiment, which allowed for the release of the rest of the encapsulated drug. This initial burst release can be beneficial in clinical applications. It may allow the drug to quickly target organs and tumour sites, and act against the cancer cells. In addition, a sustained release phase is desirable, in order to ensure a high concentration of the drug over an extended period, and hence reduce the frequency of drug administrations. It is probable that in vivo naringenin-loaded polymeric micelles based on Pluronics P123 and F127 will display similar pharmacokinetic properties to those demonstrated in vitro.

**Figure 6:**
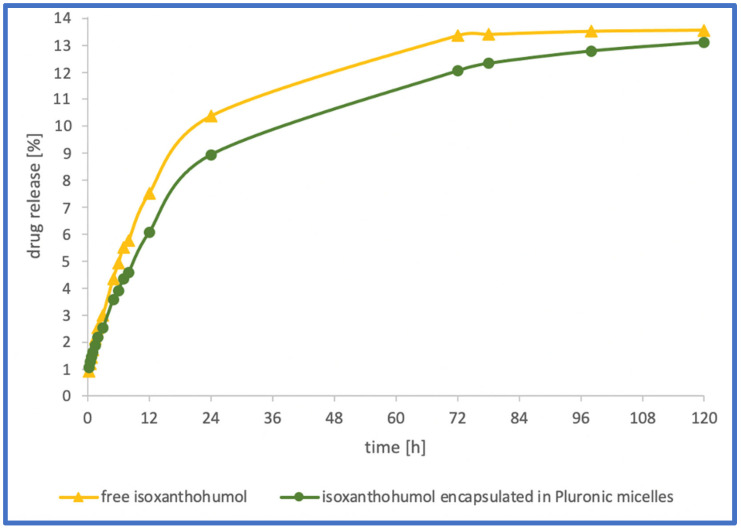
In vitro release profile of isoxanthohumol-loaded P123/F127 Pluronic micelles (ratio 1:5) and free isoxanthohumol.

From the group of xanthohumols, a sample containing isoxanthohumol was chosen from a technical point of view. The solubility in water is very low; therefore, it was more feasible to measure the release profile measurement of isoxanthohumol, which exhibits a solubility that is nearly four times higher in aqueous solutions than its isomer [33]. Investigation of the in vitro release of encapsulated isoxanthohumol revealed that polymeric micelles based on Pluronics P123 and F127 were not able to enhance its water solubility effectively. As shown in Figure 6, only 13% of the flavonoid was released after 120 h, which is similar to the 14% value obtained for crude isoxanthohumol. The differences between the release profiles of both samples were negligible, and thus it could be assumed that the encapsulation in Pluronics did not change the release behaviour of isoxanthohumol. Finally, the conclusion that can be drawn is that Pluronics P123 and F127 are not effective carriers for the delivery of isoxanthohumol. This may be a result of the high hydrophobicity of isoxanthohumol, which stems from the presence of the prenyl group in this molecule, and this hydrophobicity cannot be overcome by a designed mixed micellar system.

### 2.6. In Vitro Cytotoxicity

The Sulphorhodamine B (SRB) cytotoxicity assay performed on the human colon cell line (HT-29) revealed the IC50 values of prepared micellar formulations containing naringenin (Table 6) and xanthohumol (Table 7). IC50 is defined as the drug concentration required to induce 50% cell mortality [34]. Dose-dependent cytotoxicity of the flavonoids was observed in all cases, regardless of whether the drug was encapsulated or in a free form. The results of the in vitro cytotoxicity investigation showed that the IC50 values reached for single-type micelles of P123 and F127 were higher than 100 μg/mL, except for 1:20, which was 95.24 ± 10.35 μg/mL. Compared to the IC50 of crude naringenin which was estimated to be >100 μg/mL, the conclusion was drawn that they are not effective in drug delivery to cancer cells.

The results are more promising in the case of mixed micelle formulations, where the tendency for IC50 to decrease with an increase in the polymer content was observed. All formulations exhibited greater cytotoxic activity than crude naringenin. Based on these data, it was concluded that mixed P123/F127 micelles are effective systems for the delivery of anticancer drugs. Furthermore, the highest amount in the formulation allows for better cytotoxic activity, which can be associated with the higher water solubility of the flavonoid obtained through encapsulation in polymeric micelles. However, to completely ensure the efficiency of prepared polymeric micelles and confirm the safety of their application in a living organism, additional evaluation of the in vivo cytotoxicity were carried out in the subsequent steps of the investigation.

Based on the non-cytotoxic properties of naringenin encapsulated in single-type micelles, only mixed P123/P127 micellar formulations were chosen for the test in the case of the xanthohumol in vitro cytotoxicity assay.

The IC50 values obtained for three drug to polymer ratios, 1:5, 1:10, and 1:20, were 20.96 ± 1.81, 20.82 ± 1.19 and 22.24 ± 0.61 μg/mL, respectively. The differences between them are negligible and, unfortunately, any dependence on the polymer concentration could not be observed. Compared to the IC50 of free xanthohumol and isoxanthohumol estimated at 8.28 and 17.24 μg/mL, respectively, these formulations did not provide increased cytotoxicity against the human cancer cell line of colon adenocarcinoma (HT-29). However, it is possible that similar to the case of naringenin, a higher polymer content would provide better cytotoxic activity. Therefore, further investigation of samples with higher polymer contents should be performed, in order to determine which flavonoid to polymer ratio provides the maximum effectiveness of xanthohumols in destroying cancer cells.

## 3. Conclusions

Pluronics P123 and F127 were found to efficiently solubilise poorly water-soluble flavonoids such as naringenin, xanthohumol, and isoxanthohumol. A thin-film rehydration method of nanocarriers synthesis provided high drug loadings and encapsulation efficiencies—93.5% to 100%—for both single-type micelles of Pluronics P123 and F127, as well as their mixed counterparts. The obtained naringenin release profile indicated a significant improvement in water solubility, and thus bioavailability, of the Pluronic-encapsulated flavonoid in comparison to that of its free form. Unfortunately, the same delivery system based on Pluronics P123 and F127 was not very successful in the case of isoxanthohumol solubilisation. However, further investigation is required to clarify this mechanism.

Based on the results of the dynamic light scattering analysis presented above, it can be claimed that due to their appropriate size and relatively neutral charge, flavonoid-loaded Pluronic micelles can effectively distribute the drug into tumour tissues, which makes these nanocarriers ideal candidates for passive targeting of cancer cells by the enhanced permeation and retention (EPR) effect. The in vitro cytotoxicity of naringenin in the Pluronic formulations was investigated by the SRB assay with human colon cancer cells. The results showed that the mixed micelles provided higher cytotoxicity against the cancer cells than single-type micelles. Furthermore, the tendency to enhance the cytotoxicity with increasing polymer content in the formulation was observed. In the case of xanthohumol or isoxanthohumol, an enhanced in vitro cytotoxic activity was not achieved; however, it was associated with a lower polymer content in the tested formulations.

## 4. Materials and Methods

### 4.1. Materials

**Figure 7:**
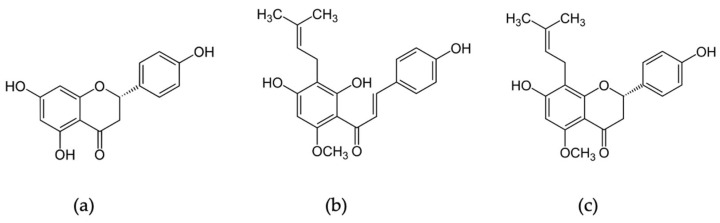
Chemical structures of the tested flavonoids: (**a**) naringenin, (**b**) xanthohumol and (**c**) isoxanthohumol.

Pluronics P123 and F127, naringenin and the salts, sodium phosphate monobasic and potassium phosphate dibasic, were purchased from Sigma-Aldrich Chemical Co. (Poznań, Poland). All solvents used in the experiments, methanol, ethanol, dimethylsulfoxide (DMSO), and a salt, sodium chloride, were acquired from Avantor Performance Materials S.A. (Gliwice, Poland). Distilled water was used in all experiments. Xanthohumol was isolated from the supercritical extraction of spent hop originating from supercritical carbon dioxide of hop variety Marynka obtained from the production of hop extracts (New Chemical Syntheses Institute, Puławy, Poland). Isoxanthohumol was obtained by isomerizing xanthohumol in 1% NaOH, according to the method described in [35]. Chemical structures of the tested flavonoids are presented in Figure 7. 

### 4.2. Nanocarriers Synthesis

Naringenin-loaded Pluronic micelles were prepared via a thin-film hydration method. Stock solutions of naringenin (2 mg/mL), Pluronic P123 (20 mg/mL) and Pluronic F127 (20 mg/mL) were prepared in methanol. The required amounts of stock solutions were placed in round bottom flasks in order to obtain different ratios of flavonoid to polymer (from 1:5 to 1:50). At first, single-type micelles of Pluronic P123 as well as Pluronic F127 with naringenin were synthesised separately. Then, formulations containing mixed micelles from Pluronics P123 and F127 were developed. In all samples, the ratio of P123 to F127 was 1:1. The solvent was then removed using a rotary evaporator (Buchi Rotavapor R-100) under reduced pressure at 50 °C and 60 rpm for 60 min to yield a drug-containing polymeric film on the wall of the flask. To ensure that residual methanol was thoroughly dried, flasks were placed in a vacuum dryer at 40 °C for about 24 h. After that, the resulting polymeric films containing naringenin were rehydrated in 60 mL of distilled water, using an ultrasound bath at 37 °C for 15–60 min, depending on the film solubility, and further sonicated in an ultrasound homogeniser (Sonics Vibra-Cell VCX 500, Sonics & Materials, Inc., Newtown, CT, USA) for the next 15 min (20 kHz, 500 W, max. temp. 40 °C) to obtain a homogeneous solution of drug-encapsulated micelles. After centrifugation at 5000 rpm for 10 min, no precipitation of non-encapsulated naringenin was observed. Subsequently, a lyophilisation process of the prepared formulations was carried out in a freeze dryer (Labconco Freezone, Labcono Corporation, Kansas City, MO, USA). Xanthohumol- and isoxanthohumol-loaded Pluronic micelles were prepared according to the analogue procedure.

### 4.3. Characterisation of Polymeric Micelles

#### 4.3.1. Encapsulation Efficiency and Drug Loading

Taking into account the poor water solubility of naringenin and xanthohumol, and the fact that no precipitation occurred after centrifugation, the amount of free flavonoid in the formulations was negligible. Accordingly, almost the total amount of flavonoids in the formulations was entrapped into the micelles.

To determine the drug loading (*DL*) and encapsulation efficiency (*EE*), a few milligrams of the lyophilised formulations were dissolved in 25 mL of flavonoid DMSO, and the amount of encapsulated flavonoid was quantified spectrophotometrically using a UV-VIS spectrophotometer (JASCO V-630) at 289 nm for naringenin and 370 nm for xanthohumol. *DL* and *EE* were calculated as per Equations (1) and (2):

(1) $$DL=\frac{mass\;of\;flavonoid\;encapsulated\;\;}{total\;mass\;of\;the\;sample\;after\;liophilization}\times 100\%$$

(2) $$EE=\frac{experimental\;DL\;\;}{theoretical\;DL\;\;}\times 100\%$$

#### 4.3.2. Particle Size Analysis

The mean particle sizes of the nanomicelles were measured via dynamic light scattering using an NICOMP 380 ZLS Particle Sizer. The same equipment was used for the Zeta potential measurement. Lyophilised nanomicelles samples were previously dissolved in distilled water, and their dissolution was assisted by 10–30 min sonification in an ultrasonic bath in order to obtain a uniform distribution of nanomicelles. Additionally, the investigation of micelle stability upon dilution was conducted using the following procedure. Briefly, samples with a concentration of 0.75 mg/mL were diluted 2, 5 and 10 times with distilled water. The results of the measurements—the mean diameter of particles and standard deviation—were compared to those obtained without dilution. All measurements were made three times.

#### 4.3.3. Differential Scanning Calorimetry (DSC) Characterisation

Differential Scanning Calorimetry analysis is applicable in the investigation of pharmaceutical formulations to detect possible interactions between the components of the prepared formulations, as well as potential changes in the physicochemical properties of both the active substance and a drug carrier [36]. Differential scanning calorimetry analysis was performed using the DSC 1 STARe System (Mettler Toledo, Columbus, OH, USA) with a Huber TC100 immersion cooler. Samples of 5.54–5.88 mg of the crude naringenin, Pluronic F127 and nanomicellar formulation of naringenin with Pluronic F127 1:10 were loaded into hermetically sealed aluminium capsules with voluminosity 40 μL. The analysis was carried out under a nitrogen flow of 60 mL/min. The following temperature program, consisting of four segments, was established:

1. Heating from 0 °C to 300 °C at a heating rate of 10 °C/min;
2. Isothermal heating for 2 min at 300 °C;
3. Cooling down from 300 °C to 0 °C at a rate of 10 °C/min;
4. Heating from 0 °C to 300 °C at a heating rate of 10 °C/min.

The DSC curves were recorded as heat flow (mW) as a function of the temperature.

#### 4.3.4. In Vitro Release of Flavonoids from Pluronic Micelles

In order to evaluate the in vitro release of flavonoids from the prepared nanomicellar formulations, a dialysis method was utilised. The experiment was carried out under physiological conditions—pH 7.4 and temperature 37 °C—that mimicked the environment of the human small intestine, where a drug is supposed to be absorbed after oral administration. The release medium was 0.01 M phosphate buffer saline (PBS) with pH 7.4—a water solution consisting of 0.80% sodium chloride, 0.14% sodium phosphate monobasic, 0.024% potassium phosphate dibasic and 0.20% potassium chloride. PBS represented the osmolality and ion concentrations of the human body fluids, and helped to maintain a constant pH. The role of a 20% addition of ethanol to the release medium was to assist in the quantification of poorly water-soluble flavonoids. Then, 90 mg of naringenin-loaded polymeric mixed micelles (Pluronic P123 and F127, naringenin:Pluronics 1:5) were dissolved in 50 mL of the release medium (PBS + ethanol 20% (*v*/*v*)), placed in a dialysis bag (molecular weight cutoff 1000 Da), then suspended in 500 mL of release medium and kept at a constant temperature of 37 °C under gentle agitation from a magnetic stirrer. At the predetermined time intervals, 5 mL samples of the release medium were withdrawn, and the same amounts of a fresh medium were added to keep the volume constant. The amount of naringenin released was determined by a UV-Vis spectrophotometer at 340 nm, and calculated from the preprepared calibration curve. The percentage of drug released (*DR*) from micelles was calculated as the ratio of naringenin quantified in the release medium at a specific time point to the amount of naringenin placed in the dialysis bag—Equation (3).

(3) $$DR=\frac{mass\;of\;flavonoid\;in\;the\;release\;medium\;}{mass\;of\;flavonoid\;added\;to\;the\;dialysis\;bag}\times 100\%\;\;\;\;\;\;$$

The release profile of the crude naringenin was prepared according to the same procedure. Subsequently, the analogous release profiles of isoxanthohumol-loaded polymeric micelles (Pluronic P123 and F127, isoxanthohumol:Pluronics 1:5) and free isoxanthohumol were determined. The amount of released isoxanthohumol was measured at a wavelength of 330 nm.

#### 4.3.5. In Vitro Cytotoxicity

Determination of IC50 by SRB method

HT-29 cells were cultured in α-MEM medium (IITD PAN, Wroclaw, Poland) with the addition of 10% bovine serum FBS (Gibco, Paisley, UK), 2 mM glutamine (Gibco, Paisley, UK) and a mixture of antibiotics (100 μg/mL penicillin, 100 μg/mL streptomycin, 0.25 μg/mL amphotericin, Gibco) at 37 °C and 5% CO_2_ in a HeraCELL incubator (Thermo Fisher Scientific, Waltham, MA, USA). The cells were passaged with a Trypsin/EDTA solution (0.05% trypsin, 0.002% EDTA, 0.85% NaCl, IITD PAN, Wrocław, Poland). Cells that detached from the medium with trypsin/EDTA solution were counted on a Burker camera, and the cell suspension was prepared in a complete culture medium at a concentration of 5 × 10^4^ cells/mL. They were then sown at 5 × 10^3^ cells/well into a 96-well plate (Sarstedt, Germany). The next day, the starting solution of the test compound (stock) was prepared at a naringenin concentration of 2.5 mg/mL at a concentration of 0.9% NaCl, followed by a dilution series in the whole culture medium (100 μg/mL, 75 μg/mL, 50 μg/mL, 25 μg/mL, 10 μg/mL, 5 μg/mL, 1 μg/mL). The medium from the 96-well plate was removed, and 100 μL per well of the test compound solution was added in 4 replicates for each dilution. Cells with test compounds were incubated for 48 h. After this time, the medium was removed, and fresh culture medium was added without supplements. Then, 50 μL of a 50% trichloroacetic acid solution was added to each well. The plates were incubated at 4 °C for 1 h to precipitate the proteins. The solution was removed, and the plate was rinsed 5 times with tap water. After drying with a cold stream of air from a hair dryer, 50 μL of sulphorhodamine B solution (0.4% SRB, 1% CH_3_COOH, Sigma-Aldrich Co., St. Louis, MO, USA) was added to each well and incubated in the dark for 0.5 h. The sulphorhodamine solution was removed, and the wells were rinsed four times with a 1% CH_3_COOH solution. After drying the plate, 150 μL of a 10 mM Tris-HCl solution was added to each well and incubated for half an hour, with stirring to completely dissolve the sulphorhodamine B. Absorbance measurements were taken at a wavelength of 570 nm, blanked on a Victor 2 plate reader (Perkin Elmer). IC50 values were determined from graphs of cell mortality (AK-AB)/AK versus log10 (test compound concentrations), where AK represented the absorbance for the control sample (cells growing in a complete culture medium), AB represented the absorbance of the test sample (cells growing in the medium with the addition of test compound at a specified concentration).

## Figures and Tables

**Table 1:** Results of naringenin-loaded micellar nanoparticles preparation. LE—lyophilisation efficiency, DL_theor_—theoretical drug loading, DL_exp_—experimental drug loading, EE—encapsulation efficiency.

Naringenin to Pluronic Ratio		m_added_ [mg]			m_after_ _lyophilisation_ [mg]	LE [%]	Drug Loading [%]		Encapsulated Naringenin		
		Naringenin	P123	F127			DL_theor_	DL_exp_	C [mg/L]	m [mg/g]	EE [%]
**P123**	**1:5**	150.0	750	-	612.0	**68.0**	16.7	**16.7**	93.16	180.27	**100.0**
	**1:10**	100.0	1000	-	966.2	**87.8**	9.1	**9.1**	22.91	99.24	**100.0**
	**1:20**	50.0	1000	-	897.6	**85.5**	4.8	**4.7**	13.83	47.09	**98.9**
**F127**	**1:5**	140.0	-	700	811.4	**96.6**	16.7	**16.3**	86.55	163.30	**98.0**
	**1:10**	100.0	-	1000	1059.7	**96.3**	9.1	**9.1**	64.02	96.94	**100.0**
	**1:20**	50.0	-	1000	993.9	**94.7**	4.8	**4.8**	23.27	50.16	**100.0**
**P123/F127**	**1:5**	200.0	500	500	1082.2	**90.2**	16.7	**16.7**	71.74	175.83	**100.0**
	**1:10**	100.0	500	500	994.6	**90.4**	9.1	**8.6**	40.36	86.23	**94.9**
	**1:20**	50.0	500	500	990.5	**94.3**	4.8	**4.8**	20.08	51.23	**100.0**
	**1:30**	50.0	750	750	1281.3	**82.7**	3.2	**3.2**	13.59	35.77	**100.0**
	**1:50**	20.0	500	500	880.5	**86.3**	2.0	**2.0**	10.80	23.47	**100.0**

**Table 2:** Results of xanthohumol-loaded micellar nanoparticles preparation. LE—lyophilisation efficiency, DLtheor—theoretical drug loading, DLexp—experimental drug loading, EE—encapsulation efficiency.

Xanthohumol to Pluronic Ratio		m_added_ [mg]			m_after_ _lyophilisation_ [mg]	LE [%]	Drug Loading [%]		Encapsulated Xanthohumol		
		Xanthohumol	P123	F127			DL_theor_	DL_exp_	C [mg/L]	m [mg/g]	EE [%]
**P123/F127**	1:5 *	116.0	300	300	615.4	**85.9**	16.2	**15.2**	29.58	151.52	**93.5**
	**1:10**	64.0	320	320	485.3	**68.9**	9.1	**9.1**	18.70	99.05	**100.0**
	**1:20**	33.0	340	340	533.0	**74.8**	4.6	**4.6**	10.72	51.64	**100.0**

* sample containing isoxanthohumol.

**Table 3:** Size of the naringenin-loaded polymeric micelles dissolved in water. ξ—Zeta potential, σ—standard deviation, PDI—polydispersity index.

	P123				F127				P123/F127			
	ξ [mV]	Size [nm]	σ [nm]	PDI	ξ [mV]	Size [nm]	σ [nm]	PDI	ξ [mV]	Size [nm]	σ [nm]	PDI
**1:5**	8.13	**229.4** ± 0.7	142.2	0.38	precipitate	**38.2** ± 0.4	19.5	0.26	5.96	**138.1** ± 2.4	73.15	0.28
**1:10**	−0.03	**56.9** ± 0.4	35.6	0.39	precipitate	**27.5** ± 0.3	12.9	0.22	−8.88	**47.1** ± 0.0	27.0	0.33
**1:20**	4.31	**48.8** ± 0.2	30.5	0.39	0.46	**29.8** ± 0.1	14.2	0.23	−4.85	**38.0** ± 0.2	19.3	0.26
**1:30**	-	-	-	-	-	-	-	-	3.44	**47.1** ± 0.0	27.0	0.33
**1:50**	-	-	-	-	-	-	-	-	2.75	**44.1** ± 0.2	24.4	0.31

**Table 4:** Size of the xanthohumol-loaded polymeric micelles dissolved in water. ξ—Zeta potential, σ—standard deviation, PDI—polydispersity index.

	P123/F127			
	ξ [mV]	size [nm]	σ [nm]	PDI
1:5 *	−4.26	**30.5** ± 0.2	17.9	0.34
**1:10**	4.26	**30.5** ± 0.1	18.0	0.35
**1:20**	−3.15	**30.4** ± 0.1	17.9	0.35

* sample containing isoxanthohumol.

**Table 5:** Changes of naringenin-loaded particle size against dilution with distilled water.

Dilution	0	×2	×5	×10
**1:5 P123**	229.4 ± 0.7 nm	217.0 ± 1.8 nm	171.9 ± 0.2 nm	36.3 ± 0.2 nm
**1:5 P123/F127**	138.1 ± 2.5 nm	142.0 ± 5.8 nm	155.1 ± 6.6 nm	139.2 ± 4.6 nm

**Table 6:** Values of IC50 for naringenin encapsulated in polymeric micelles with different drug to Pluronic ratios.

Naringenin to Pluronic Ratio	P123 IC_50_ [μg/mL]	F127 IC_50_ [μg/mL]	P123/F127 IC_50_ [μg/mL]
**1:5**	>100	>100	59.03 ± 5.77
**1:10**	>100	>100	55.03 ± 5.02
**1:20**	95.24 ± 10.35	>100	54.13 ± 11.84
**1:30**	-	-	32.51 ± 1.67
**1:50**	-	-	23.44 ± 1.43

**Table 7:** Values of IC50 for xanthohumol encapsulated in polymeric micelles with different drug to Pluronic ratios.

Xanthohumol to Pluronic Ratio	P123/F127 IC_50_ [μg/mL]
1:5 *	20.96 ± 1.81
**1:10**	20.82 ± 1.19
**1:20**	22.24 ± 0.61

* sample containing isoxanthohumol.

## Data Availability

The data, if not directly presented in this study, are available on request from the corresponding author.
